# Characterization of Anti-seizure Medication Treatment Pathways in Pediatric Epilepsy Using the Electronic Health Record-Based Common Data Model

**DOI:** 10.3389/fneur.2020.00409

**Published:** 2020-05-12

**Authors:** Hunmin Kim, Sooyoung Yoo, Yonghoon Jeon, Soyoung Yi, Seok Kim, Sun Ah Choi, Hee Hwang, Ki Joong Kim

**Affiliations:** ^1^Department of Pediatrics, Seoul National University Bundang Hospital, Seongnam, South Korea; ^2^Healthcare ICT Research Center, Seoul National University Bundang Hospital, Seongnam, South Korea; ^3^Department of Pediatrics, Ewha Woman's University Medical Center, Seoul, South Korea; ^4^Department of Pediatric, Seoul National University Children's Hospital, Seoul, South Korea

**Keywords:** Common Data Model (CDM), treatment pathway, epilepsy, antiseizure medications, drug-resistant epilepsy

## Abstract

The purpose of this pilot study was to analyze treatment pathways of pediatric epilepsy using the common data model (CDM) based on electronic health record (EHR) data. We also aimed to reveal whether CDM analysis was feasible and applicable to epilepsy research. We analyzed the treatment pathways of pediatric epilepsy patients from our institute who underwent antiseizure medication (ASM) treatment for at least 2 years, using the Observational Medical Outcomes Partnership (OMOP)-CDM. Subgroup analysis was performed for generalized or focal epilepsy, varying age of epilepsy onset, and specific epilepsy syndromes. Changes in annual prescription patterns were also analyzed to reveal the different trends. We also calculated the proportion of drug-resistant epilepsy by applying the definition of seizure persistence after application of two ASMs for a sufficient period of time (more than 6 months). We identified 1,192 patients who underwent treatment for more than 2 years (mean ± standard deviation: 6.5 ± 3.2 years). In our pediatric epilepsy cohort, we identified 313 different treatment pathways. Drug resistance, calculated as the application of more than three ASMs during the first 2 years of treatment, was 23.8%. Treatment pathways and ASM resistance differed between subgroups of generalized vs. focal epilepsy, different onset age of epilepsy, and specific epilepsy syndromes. The frequency of ASM prescription was similar between onset groups of different ages; however, phenobarbital was frequently used in children with epilepsy onset < 4 years. Ninety-one of 344 cases of generalized epilepsy and 187 of 835 cases of focal epilepsy were classified as medically intractable epilepsy. The percentage of drug resistance was markedly different depending on the specific electro-clinical epilepsy syndrome [79.0% for Lennox-Gastaut syndrome (LGS), 7.1% for childhood absence epilepsy (CAE), and 9.0% for benign epilepsy with centrotemporal spikes (BECTS)]. We could visualize the annual trend and changes of ASM prescription for pediatric epilepsy in our institute from 2004 to 2017. We revealed that CDM analysis was feasible and applicable for epilepsy research. The strengths and limitations of CDM analysis should be carefully considered when planning the analysis, result extraction, and interpretation of results.

## Introduction

Epilepsy is a heterogeneous and complex brain disorder comprising of many seizure types and epilepsy syndromes (1, 2). It is common practice for physicians to begin long-term, daily anti-seizure medication (ASM) treatment after a patient has experienced unprovoked seizures (3). The ASMs lead to satisfactory control of seizures for as many as 60–70% of newly treated patients (4). Unfortunately, 20–30% of patients have uncontrolled epilepsy (drug-resistant epilepsy) with seizures, adverse effects, and significantly increased risk of mortality and morbidity (1, 4). An analysis of ASM treatment patterns in a real clinical practice will help identify various types of patients with epilepsy.

The treatment choice for epilepsy is empirical and often based on trial and error (1). Since the 1980s more than (>) 15 ASMs have been introduced, giving rise to more choices in selecting the first drug for epilepsy treatment but making it more difficult to make optimal treatment decisions (1). Patients who fail to improve with the first drug continue treatment with an alternative monotherapy (substitution) or a combination therapy (add-on), where a second drug is added to the current monotherapy (1, 4, 5). Regarding treatment choices for pediatric epilepsy, research findings are limited, and many clinical questions remain unresolved; thus, physicians must often rely on clinical judgement (6). The available information about the treatment sequence for pediatric syndromes and the use of therapy in actual medical practice is also limited (6). In an effort to achieve consensus on a number of treatment options, 41 U.S. experts were surveyed on pediatric epilepsy and seizures, including opinions regarding 645 treatment options, and the overall recommendations were reported in 2015 (6). Although the experts reached consensus on many treatment options, it remains to be evaluated whether treatment recommendations reflect actual practice and whether there is any difference between actual practice and recommendations.

A study calculating ASM utilization patterns for patients with epilepsy has been conducted using the Swedish total-population register data (7). The study reported a limited number of drug choices for the treatment of the majority of new patients (7). Studies on patterns of drug utilization using electronic health record (EHR) and claims data from various countries can be found in the Observational Health Data Sciences and Informatics (OHDSI) community (8, 9). OHDSI is an international collaborative aiming at creating open-source solutions that emphasize the value of observational health data through large-scale analytics^1^. OHDSI adopts a distributed research network with federated data harmonized to the Observational Medical Outcomes Partnership Common Data Model (OMOP-CDM), which represents healthcare data from diverse sources in a consistent and standardized way (10). In the CDM, domains such as patient demographics, conditions, drug exposures, measurements, observations, and procedures are modeled in a patient-centric relational data model with standard vocabularies. The common data structure (format) and the common representation of the content of data record enable the development of standard analysis tools. The CDM has been introduced to generate scientific evidence from a variety of data sources and conduct large-scale collaborative researches across different data sources. Patient privacy is maintained with de-identification of data sources and a distributed data model. An earlier research of the OHDSI network on characterizing treatment pathways for type 2 diabetes mellitus, hypertension, and depression revealed heterogeneity of the pathways among different data sources (8).

An assessment of the current treatment choices for pediatric epilepsy using CDM data will provide real-word evidence of common practice and treatment patterns as well as current unmet needs for the development of more effective ASMs. Here, we aimed at conducting population-level analysis to characterize real-world pathways for the treatment of pediatric patients with epilepsy during the first 2 years of therapy using the CDM. The main purpose of this pilot study was to show the applicability of the CDM in big data analysis for epilepsy. The primary goal underlying the feasibility of our approach was to understand whether CDM analysis in epilepsy can provide the percentage of drug-resistant epilepsy in our pediatric cohort. We also aimed to provide detailed information regarding ASM prescription data in different types of epilepsy, age groups, and trends for each year between 2004 and 2017.

## Materials and Methods

### Study Design

This is a retrospective, observational study. We analyzed ASM prescription patterns of all pediatric epilepsy patients in our hospital, whose treatment lasted longer than 2 years, from initial diagnosis to end of treatment. The main question in this study was whether we can produce the exact number of patients with drug-resistant epilepsy by applying the recent International League Against Epilepsy (ILAE) definition (11) using the CDM. To calculate this number, we analyzed the total number of ASMs used during the treatment period. Since ASM withdrawal is considered after at least 2 years of seizure freedom (12, 13), we selected patients whose follow-up period was longer than 2 years. In our analysis, we defined drug resistance as an add-on of a third medication of the course of treatment. Further analysis of the treatment pathway and the number of ASMs used was performed by epilepsy category (focal epilepsy vs. generalized epilepsy) and onset age (under 4 years; between 4 and 13 years; over 13 years). We also performed analysis of distinct epilepsy syndromes [benign epilepsy with centrotemporal spikes (BECTS), childhood absence epilepsy (CAE), and Lennox-Gastaut syndrome (LGS)] that show typical drug resistance profiles. We analyzed the pattern of annual ASM prescription for monotherapy to find whether CDM analysis can provide information regarding varying tendency of ASM prescription for each year of treatment. Finally, we examined the serial changes in the number of people treated with certain ASMs in a specific year and the proportion of specific ASMs for each year of treatment.

### Population

The population of interest was defined as pediatric patients with epilepsy who had at least 2 years of continuous observation and persistent treatment following initiation.

The initial event cohort included patients who had had exposure to ASMs for the first time in the person's history at age ≤ 18, observation of at least 0 days prior and 730 days after the event index date to include at least 2 years prescription data, and limited initial events to the earliest event per person. The index date was the time of first exposure to the ASM class of N03A code defined in the Anatomical Therapeutic Chemical (ATC) Classification System. Specifically, patients had to fulfill the following criteria: at least one prescription of an ASM exposure between 121 and 240 days, 241 and 360 days, 361 and 480 days, 481 and 600 days, and 601 and 730 days after the event index date (first prescription of ASM) (see Table S1 for epilepsy diagnosis codes).

Additional qualifying inclusion criteria were that the patient had been diagnosed with epilepsy at least once, had at least one epilepsy diagnosis before inclusion in the study, the epilepsy diagnosis had started between all days before and all days after the event index date, and the patient had had regular follow-ups (see Table S2 for epilepsy drugs codes). The purpose of this inclusion was to prevent missing the patients whose epilepsy diagnosis was missed in any visit.

**Figure 1:**
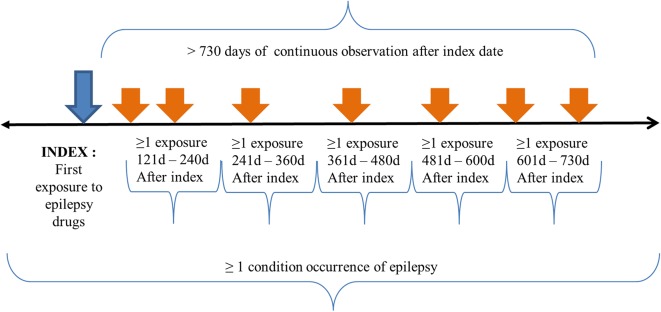
Treatment pathway population criteria. The index date for our study population was the time of first exposure to ASMs for the first time in a patient's history with age ≤ 18 years. The patient had to have continuous observation for at least 730 days after the event index date and limited initial events to the earliest event per person. The patients had to have at least one epilepsy diagnosis code between all days before and after the event index date. The patients had to have a regular follow-up for at least 2 years after the index date. Specifically, patients had to have a drug exposure to epilepsy drugs in each 120 day period after the index date.

The end date strategy was not selected. By default, the cohort end date was the end of the observation period that contained the index event. Without a limitation in the observation period before the index date, the patient had to have been observed for at least 730 days after the index date. The patient had to have at least one condition occurrence of epilepsy listed in the diagnosis code between all days before the index date and all days after the index date. Regarding the definition of persistent treatment, the patient had to have at least one occurrence of exposure to ASMs in each 120 day period during 2 years after the index date. The cohort meeting the population inclusion and exclusion criteria is depicted in Figure 1.

### Data Sources

The over 14 year-old EHR data of Seoul National University Bundang Hospital (SNUBH), a tertiary university hospital in the metropolitan area, contain > 1.8 million patients. Data from May 2003 through October 2017 were transformed into an OMOP CDM, version 5.2. Since the data sources were de-identified, this study was approved with waiver of informed consents or exemption by the institutional review boards at our institution (IRB No: X-1810/497-902).

### Open Source Treatment Pathway

In this study, the OHDSI's open-source software treatment pathway was used^2^. For each patient in the cohort, this software identifies the sequence of treatments in the RxNorm ingredient level (8). Once each patient's treatment sequence is constructed, the number of unique patients with the same treatment sequence is calculated. The counts by the index year of the first exposure is also stratified for further analysis. A limitation of this code is that the treatment pathway does not distinguish between switching and adding medications (8). The sequences were limited to 20 medications (8).

### Statistical Methods

This is a descriptive summary analysis of data exploration and characterization with no specified assumptions; as such, no statistical analysis was performed.

## Results

### Participants

We identified 1,192 patients [male: female, 653:539; mean age at diagnosis, 8.3 ± 5.0 years (range: 3.3–13.3 years)] who had > 2 years follow-up (mean, 6.5 ± 3.2 years; range: 2.0–14.2 years) and ASM prescription data after the CDM query application. Patients' available specific diagnosis for epilepsy is shown in Table 1.

**Table 1:** Epilepsy classification and types, common data model (CDM) concept identification (ID), and number (*n*) of patients with specific epilepsy diagnosis and classification.

**CDM concept ID**	**Electronic health record (EHR) source name**	***n***
	Focal Epilepsy	835
374915	Localization-related epilepsy, not otherwise specified	613
4185733	Benign epilepsy with centrotemporal spikes	67
4102345	Temporal lobe epilepsy	49
4044080	Childhood occipital epilepsy (Panayiotopoulos type)	44
4047888	Frontal lobe epilepsy	26
4046207	Occipital lobe epilepsy (Gastaut type)	24
4043551	Epilepsy with continuous spike wave during slow-wave sleep	4
4044084	Supplementary motor area epilepsy	4
4046206	Parietal lobe epilepsy	3
4041672	Rasmussen syndrome	1
	Generalized Epilepsy	344
4055361	Generalized epilepsy, not otherwise specified	137
4179936	Childhood absence epilepsy	56
376105	West syndrome	44
4046213	Lennox-Gastaut syndrome	38
4267274	Juvenile myoclonic epilepsy	34
4046210	Juvenile absence epilepsy	15
4047897	Epilepsy with grand mal seizures on awakening	10
4043413	Myoclonic astatic epilepsy	5
4044225	Myoclonic absence epilepsy	5
	Unclassified	12

### Overall Treatment Pathway

**Figure 2:**
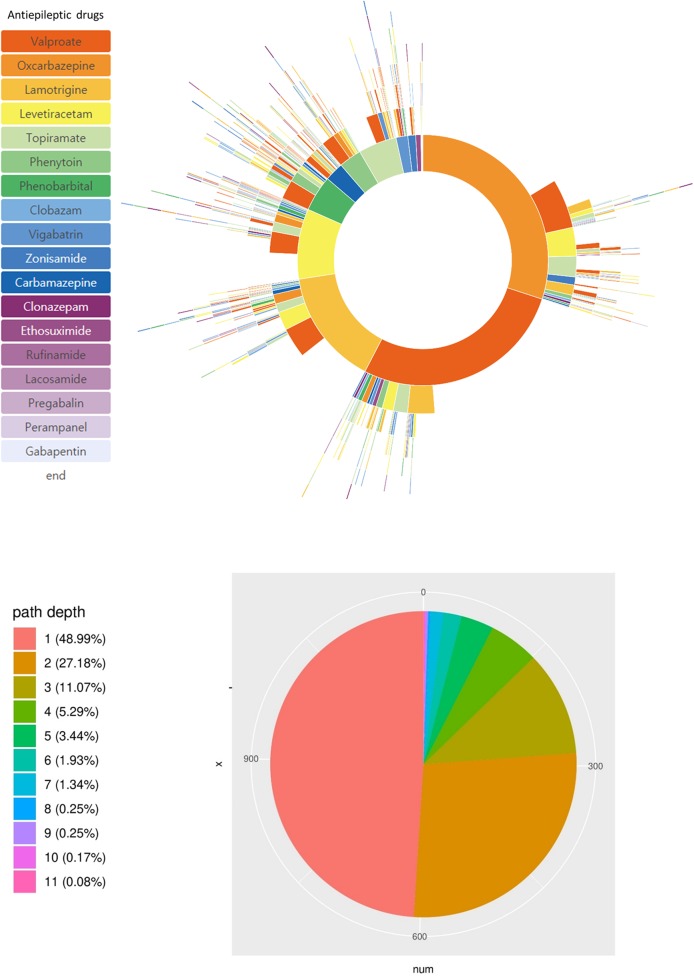
Treatment pathways of all 1,192 pediatric epilepsy patients. Specific ASMs used and their sequence is shown in the sunburst plot. The pie chart shows the number of patients with the number of ASMs used during their initial 2 year treatment.

There were 313 distinct treatment pathways in the pediatric epilepsy cohort of our hospital. The most frequently used ASMs in our cohort were valproic acid (26.4%), oxcarbazepine (17.5%), lamotrigine (13.1%), levetiracetam (11.5%), and topiramate (9.2%). During the treatment period, patients were treated with only one (49.0%) or two (27.2%) ASMs (Figure 2). We estimated that 23.8% of our epilepsy patients were drug-resistant by the definition used in our CDM analysis query in Figure 1.

### Prevalence of Treated Epilepsy and Drug-Resistant Epilepsy (DRE)

**Figure 3:**
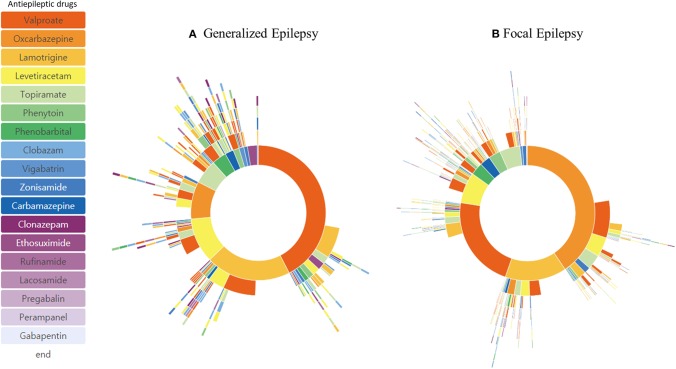
Treatment pathways of generalized epilepsy patients **(A)** and focal epilepsy patients **(B)**.

A detailed diagnosis of the epilepsy type was available in 1,179 patients (Table 1). Patients were grouped as having generalized epilepsy (344 patients) and focal epilepsy (835 patients). Patients' treatment pathway showed a distinctive pattern of ASM selection. The order of the three most frequently used ASMs for focal epilepsy was valproic acid (24.5%), oxcarbazepine (23.8%), and lamotrigine (13.4%), whereas for generalized epilepsy the order was valproic acid (31.8%), lamotrigine (17.1%), and levetiracetam (12.7%) (Figure 3). The percentage of ASM resistance was 26.5% for generalized epilepsy and 22.4% for focal epilepsy.

### Subgroup Analysis

**Figure 4:**
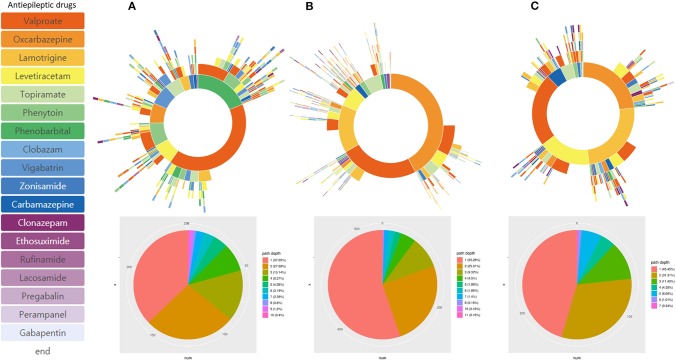
Treatment pathways according to different onset age of epilepsy. **(A)** Onset < 4 years-old, **(B)** onset age: 4–13 years-old, and **(C)** Onset > 13 years-old.

**Figure 5:**
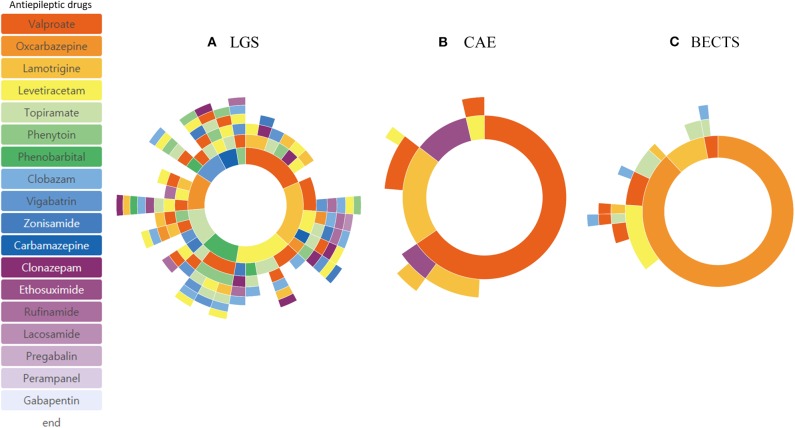
Treatment pathways of different epilepsy syndromes: Lennox-Gastaut syndrome (LGS) **(A)**, Childhood Absence Epilepsy (CAE) **(B)**, and Benign Epilepsy with Centro-Temporal Spikes (BECTS) **(C)**.

Subgroup analysis based on different age of onset showed distinctive treatment pathways (Figure 4). Phenobarbital was one of the frequently used ASMs in patients with epilepsy onset < 4 years of age. The frequency of lamotrigine, oxcabazepine, valproic acid, and levetiracetam prescription was similar in patients with epilepsy onset > 13 years of age. Drug resistance differed at 33.1% (onset age, < 4 years), 20.0% (onset age, 4–13 years), and 23.2% (onset, >13 years). In addition, we identified three distinctive pediatric epilepsy syndromes in each subgroup analysis, which showed a remarkably different treatment pathway (Figure 5). Drug resistance was 79.0% for LGS, 7.1% for CAE, and 9.0% for BECTS.

### Annual Trend of ASM Prescription

**Figure 6:**
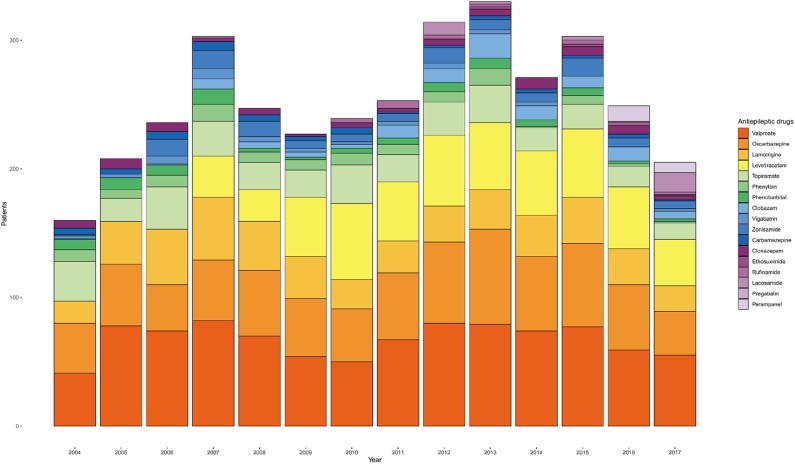
Annual trend of anti-seizure medication (ASM) prescriptions for monotherapy from 2004 to 2017.

The annual prescription volume for certain ASMs is shown in Figure 6. The annual changes of the actual ASM prescription number for patients who underwent monotherapy for a specific year is visualized in a single plot. The number of patients on valproic acid had decreased after issues in adolescent women (14, 15), but in recent years, it has increased again. We also identified a decrease in the use of topiramate since 2013. Use of levetiracetam has increased steadily after its approval in our country since 2007 (16). A slight increase in rufinamide and perampanel prescriptions was also observed after introduction of these drugs in the Republic of Korea.

## Discussion

### Applicability of CDM Analysis for Drug Resistance Evaluation

The prevalence of drug-resistant epilepsy ranges from 20 to 40% and varies depending on the definition and population. Up to 20% of pediatric epilepsy patients show drug resistance (17–19), and this increases up to 30 or 40% in the adult population (20–22). The most recent systematic review and meta-analysis reported that the pooled prevalence of drug-resistant epilepsy in newly diagnosed epilepsy patients was 25% (95% confidence interval: 17–32%) (23). We applied the CDM analysis to estimate the proportion of DRE in a cohort of all pediatric epilepsy patients in our institution using the ILAE criteria for DRE (11). The proportion of patients who received >3 ASMs during their first 2 year treatment was 24.4%, which is concordant with previous reports of long-term follow-up studies (17–19). A study that applied the same definition revealed that from 508 pediatric patients, 87 patients (19%) were drug-resistant (24).

We calculated the proportion of DRE in our pediatric epilepsy cohort by using a surrogate search condition of the number of ASMs used; the results were concordant with previous reports. Thus, we revealed that CDM analysis is both feasible and applicable in certain fields of epilepsy research. In addition to the number of ASMs used during treatment, detailed prescription information became available with this analysis. Information of overall ASM prescriptions is available with current hospital information data-structure, but it does not provide data based on individual patients. Analysis of ASM prescription data and different treatment pathway data can be helpful for evaluating the real-life treatment and practice.

### Proportion of DRE in Various Conditions and Subgroup Analysis-Feasibility of CDM Analysis in Epilepsy

The proportion of DRE of various subgroups was also available from previous studies. The proportion of DRE in focal epilepsy was similar to that in generalized epilepsy. This may be because many focal epilepsies in childhood show a benign course and good response to ASMs (25). Subgroup analysis of different infant and toddler age groups, school-age children, and adolescents showed different results in DRE and ASM selection in that DRE was more prevalent in infants and toddlers and phenobarbital was one of the commonly used ASMs in this group. This reflects the varying degree of age-specific pediatric epilepsy syndromes and age-specificity in ASM selection. Subgroup analysis of specific epilepsy syndromes such as the LGS, CAE, and BECTS revealed typical drug-resistance profiles. Most patients with LGS, except for one, used three or more ASMs, showing that LGS is drug-resistant in almost all cases. Patients with CAE were well-controlled with one or two ASMs in most cases. The few cases with BECTS represent an atypical progression reported recently (26). Subgroup analysis with the CDM in our cohort was also feasible and provided useful information regarding each subgroup.

### Treatment Pathway Analysis in Pediatric Epilepsy

We provided detailed data regarding ASM usage in our institution for a 14 year-period using the CDM. This provides extensive information such as selection of a certain ASM or different prescription patterns, which draw significant social and medical interest. Prescription of valproate for focal epilepsy is such a finding. Valproic acid is a broad spectrum ASM, most commonly used in adults with generalized onset seizures or generalized epilepsy (27). However, we found that valproate was most frequently prescribed in our cohort instead of oxcarbazepine. This may reflect the fact that valproate is considered as an initial ASM for certain focal epilepsy syndromes in guidelines and expert consensus regarding pediatric epilepsy (28, 29). A limitation in safety profiles and efficacy data in pediatric epilepsy may also underline this finding (30–32). A further regional or national study using the CDM can provide patterns of ASM prescription in South Korea. The interpretation of various different reasons can contribute in better understanding of ASM treatment in epilepsy.

### Treatment Data for Each Year or Certain Periods

In addition to the number of ASMs used during treatment, the annual changes in detailed ASM prescription information became available using CDM analysis. This type of data is not easily available in clinical practice and it is difficult to retrieve prescription data for a specific disease. We could recognize the trends in ASM prescription, which varied according to changes in guidelines, practice parameters, introduction of new ASMs, or our regional state of availability. We revealed that the analysis of annual changes using the CDM can be helpful for identifying the detailed trends in ASM prescriptions.

### Advantage of CDM Analysis

The strength of CDM analysis is that it is suitable for reviewing big data. We had 1,832 pediatric epilepsy patients from 2003 to 2017 and our cohort included 1,192 patients. We would have had to review 2 years follow-up duration of medical records of 1,192 patients to estimate the proportion of DRE and to retrieve the ASM usage if we had used the traditional method of medical record review, which is extremely labor-intensive and prone to human errors. Unlike traditional medical record review research, CDM analysis provides the flexibility of modifying search conditions and adding more data easily. Another strength of CDM research is the application in distributed network research using a large network of health databases (10) Since we have proved the feasibility of this approach, we are now planning to apply this methodology to the adult epilepsy population of our institution and to all other hospitals in our country that also use OMOP-CDM. Our research findings confirmed the applicability and feasibility of CDM analysis and suggest the possibility of a future CDM distributed network research in the field of epilepsy.

### Limitations and Important Issues in CDM Analysis

There are certain limitations to the CDM research, which should be carefully considered. We performed the analysis by forming a hypothesis, constructing a query, and searching for standard concepts to retrieve data, and comparing the data with historical cohorts. Since the de-identified CDM analysis used in this study does not review the medical record of an individual patient, specific situations, such as change of medication due to serious side effects, were not considered. Exact and precise diagnosis is critical when including patients with epilepsy and for further subgroup analysis. This is also important for incidence and risk factor studies that could utilize CDM analysis in a population database (33). Defining search concepts and constructing an adequate query is a critical part in CDM analysis. We performed multiple analyses and reviewed whether a correct cohort was constructed when identifying the cohort of our pediatric epilepsy patients with serial follow-up and ASM treatment. The cohort populations differed significantly as the query structure and contents changed. Definition of drug-resistance (11) is another limitation in this study. It is not clear and widely accepted definition. We could not retrieve data regarding seizure intervals, reasons or mode (substitution or addition) for medication change. Hence, application and validation of drug-resistance need careful interpretation.

The limitation of CDM research is also the limitation of this study. These limitations should be carefully considered when designing and interpreting research using CDM. As this study used EHR CDM data, no wash-out period was set. Because a patient's previous prescription history cannot be known in EHR data, the study subject may not be the first epilepsy patient diagnosed. Medical research utilizing big data and well-structured databases can be well-performed with CDM analysis, whereas research requiring a detailed and variable information of a small number of patients can be performed with the traditional medical record review.

### CDM Tool for Analysis

Using the open-sourced OHDSI tool that creates the treatment pathway for type 2 diabetes mellitus, hypertension, and depression from CDM data (8), we studied the treatment pathway and sub-phenotyping of ASMs in pediatric patients. The benefits of the standardized structure and the standard terminology of OMOP-CDM enable the reproduction of research with standardized analytics tools and rapid application and expansion of new research topics. It is expected that these tools will assist in analyzing the treatment patterns of drugs in rare as well as chronic diseases. CDM data can also be used to identify patterns of market penetration of medications, such as investigating the country-specific pattern use of antidiabetic medications (9). Since studies on the patterns of ASM medication use have not been reported, CDM data may be extended to investigate differences in medication use across countries with standardized analytics tools. Furthermore, CDM data may be used to examine the effects of different ASM treatment patterns on the treatment outcome in pediatric epilepsy patients.

## Conclusion

This is a unique study on the use of the CDM for evaluating epilepsy outcomes. Our pilot study on a pediatric epilepsy cohort from our entire institute showed that CDM analysis is feasible and applicable in epilepsy research. Based on this data, we can further plan distributed network research. The strengths and limitations of the research using the CDM should be carefully considered when designing the process and interpreting the study results.

## Data Availability Statement

All datasets generated for this study are included in the article/Supplementary Material.

## Ethics Statement

The studies involving human participants were reviewed and approved by Seoul National University Bundang Hospital IRB. Written informed consent for participation was not provided by the participants' legal guardians/next of kin because: IRB confirmed the waiver of consent.

## Author Contributions

HK, SYo, HH, and KK conceived and designed the analysis. SK, SYi, SC, and YJ collected the data and performed the analysis. HK, SYo, SK, HH, and SC wrote the paper.

## Conflict of Interest

The authors declare that the research was conducted in the absence of any commercial or financial relationships that could be construed as a potential conflict of interest.

## Footnotes

^1^[Dataset] OHDSI (2018). Available online at: http://www.ohdsi.org (accessed August 17, 2018).

^2^[Dataset] Treatment pathways analytics tool. Available online at: https://github.com/OHDSI/StudyProtocols/tree/master/Study%201%20-%20Treatment%20Pathways (2018) (accessed August 17, 2018).

## Abbreviations

ASM: anti-seizure medication
BECTS: benign epilepsy with centrotemporal spikes
CAE: childhood absence epilepsy
CDM: common data model
DRE: drug-resistant epilepsy
EHR: electronic health record
LGS: Lennox-Gastaut syndrome
OHDSI: Observational Health Data Sciences and Informatics
OMOP-CDM: Observational Medical Outcomes Partnership Common Data Model.
