# Supplementary material for: Characterization of Anti-seizure Medication Treatment Pathways in Pediatric Epilepsy Using the Electronic Health Record-Based Common Data Model
Source: Front Neurol. 2020 May 12;11:409. doi: 10.3389/fneur.2020.00409 (PMC7235379; doi:10.3389/fneur.2020.00409)
Supplement: Supplementary file 1 [file Table_1.DOCX]

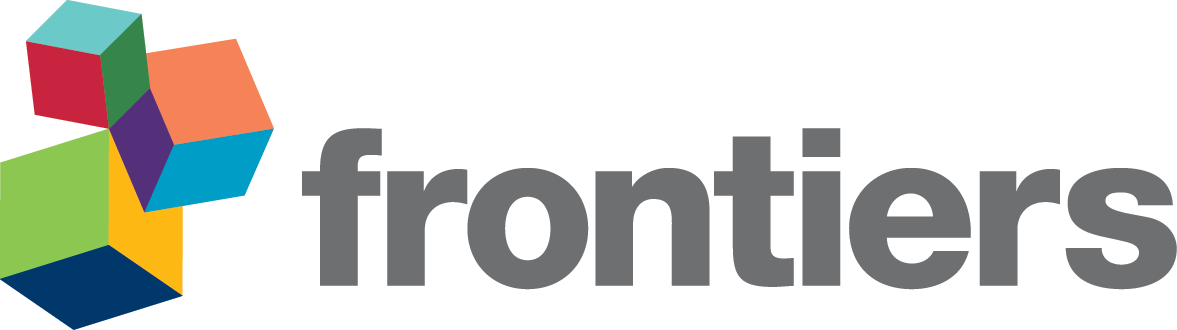


*Supplementary Material*

1. **SUPPLEMENTARY DATA**
   1. **Tables**

**Table S1.** epilepsy diagnosis

| **Concept Id** | **Concept Name** | **Domain** | **Vocabulary** | **Excluded** | **Descendants** | **Mapped** |
| --- | --- | --- | --- | --- | --- | --- |
| 4185733 | Benign Rolandic epilepsy | Condition | SNOMED | NO | NO | NO |
| 4048424 | Benign focal epilepsy of childhood | Condition | SNOMED | NO | NO | NO |
| 4179936 | Childhood absence epilepsy | Condition | SNOMED | NO | NO | NO |
| 4044080 | Childhood epilepsy with occipital paroxysms | Condition | SNOMED | NO | NO | NO |
| 380378 | Epilepsy | Condition | SNOMED | NO | NO | NO |
| 4046218 | Epilepsy undetermined whether focal or generalized | Condition | SNOMED | NO | NO | NO |
| 4043551 | Epilepsy with continuous spike wave during slow-wave sleep | Condition | SNOMED | NO | NO | NO |
| 4047897 | Epilepsy with grand mal seizures on awakening | Condition | SNOMED | NO | NO | NO |
| 4047888 | Frontal lobe epilepsy | Condition | SNOMED | NO | NO | NO |
| 374924 | Generalized convulsive epilepsy | Condition | SNOMED | NO | NO | NO |
| 4055361 | Generalized epilepsy | Condition | SNOMED | NO | NO | NO |
| 4044222 | Hemiplegia-hemiconvulsion-epilepsy syndrome | Condition | SNOMED | NO | NO | NO |
| 4274575 | Idiopathic generalized epilepsy | Condition | SNOMED | NO | NO | NO |
| 4046210 | Juvenile absence epilepsy | Condition | SNOMED | NO | NO | NO |
| 4267274 | Juvenile myoclonic epilepsy | Condition | SNOMED | NO | NO | NO |
| 4046213 | Lennox-Gastaut syndrome | Condition | SNOMED | NO | NO | NO |
| 374915 | Localization-related epilepsy | Condition | SNOMED | NO | NO | NO |
| 4150299 | Localization-related idiopathic epilepsy | Condition | SNOMED | NO | NO | NO |
| 4044082 | Localization-related symptomatic epilepsy | Condition | SNOMED | NO | NO | NO |
| 4044225 | Myoclonic absence epilepsy | Condition | SNOMED | NO | NO | NO |
| 4043413 | Myoclonic astatic epilepsy | Condition | SNOMED | NO | NO | NO |
| 4044227 | Myoclonic epilepsy - ragged red fibers | Condition | SNOMED | NO | NO | NO |
| 4044223 | Myoclonic epilepsy of early childhood | Condition | SNOMED | NO | NO | NO |
| 4044229 | Nocturnal epilepsy | Condition | SNOMED | NO | NO | NO |
| 4046207 | Occipital lobe epilepsy | Condition | SNOMED | NO | NO | NO |
| 4046206 | Parietal lobe epilepsy | Condition | SNOMED | NO | NO | NO |
| 4043406 | Primary inherited reading epilepsy | Condition | SNOMED | NO | NO | NO |
| 4147501 | Progressive myoclonic epilepsy | Condition | SNOMED | NO | NO | NO |
| 4041672 | Rasmussen syndrome | Condition | SNOMED | NO | NO | NO |
| 4043550 | Severe myoclonic epilepsy in infancy | Condition | SNOMED | NO | NO | NO |
| 4044084 | Supplementary motor epilepsy | Condition | SNOMED | NO | NO | NO |
| 4216711 | Symptomatic generalized epilepsy | Condition | SNOMED | NO | NO | NO |
| 4102345 | Temporal lobe epilepsy | Condition | SNOMED | NO | NO | NO |
| 4232071 | Tonic-clonic epilepsy | Condition | SNOMED | NO | NO | NO |
| 376105 | West syndrome | Condition | SNOMED | NO | NO | NO |
| 44784595 | Post-cerebrovascular accident epilepsy | Condition | SNOMED | NO | NO | NO |
| 36714275 | Sudden unexpected death in epilepsy | Condition | SNOMED | NO | NO | NO |
| 4197485 | Epileptic seizure | Condition | SNOMED | NO | NO | NO |
| 4046215 | Situation-related seizures | Condition | SNOMED | NO | NO | NO |

**Table S2.** epilepsy drugs

| **Concept Id** | **Concept Name** | **Domain** | **Vocabulary** | **Excluded** | **Descendants** | **Mapped** |
| --- | --- | --- | --- | --- | --- | --- |
| 21604390 | ANTIEPILEPTICS | Drug | ATC | NO | YES | NO |
| 19050832 | clobazam | Drug | RxNorm | NO | YES | NO |

**1**
